# Emergent Endovascular vs. Open Surgery Repair for Ruptured Abdominal Aortic Aneurysms: A Meta-Analysis

**DOI:** 10.1371/journal.pone.0087465

**Published:** 2014-01-31

**Authors:** Chuan Qin, Lin Chen, Ying-bin Xiao

**Affiliations:** The Department of Cardiovascular Surgery, Xinqiao Hospital, the Third Military Medical University, Chongqing, PR China; Scuola Superiore Sant’Anna, Italy

## Abstract

**Objectives:**

To systematically review studies comparing peri-operative mortality and length of hospital stay in patients with ruptured abdominal aortic aneurysms (rAAAs) who underwent endovascular aneurysm repair (EVAR) to patients who underwent open surgical repair (OSR).

**Methods:**

The Medline, Cochrane, EMBASE, and Google Scholar databases were searched until Apr 30, 2013 using keywords such as abdominal aortic aneurysm, emergent, emergency, rupture, leaking, acute, endovascular, stent, graft, and endoscopic**.** The primary outcome was peri-operative mortality and the secondary outcome was length of hospital stay.

**Results:**

A total of 18 studies (2 randomized controlled trials, 5 prospective studies, and 11 retrospective studies) with a total of 135,734 rAAA patients were included. rAAA patients who underwent EVAR had significantly lower peri-operative mortality compared to those who underwent OSR (overall OR = 0.62, 95% CI = 0.58 to 0.67, P<0.001). rAAA patients with EVAR also had a significantly shorter mean length of hospital stay compared to those with OSR (difference in mean length of stay ranged from −2.00 to −19.10 days, with the overall estimate being −5.25 days (95% CI = −9.23 to −1.26, P = 0.010). There was no publication bias and sensitivity analysis showed good reliability.

**Conclusions:**

EVAR confers significant benefits in terms of peri-operative mortality and length of hospital stay. There is a need for more randomized controlled trials to compare outcomes of EVAR and OSR for rAAA.

## Introduction

An aortic aneurysm is defined as a permanent, localized focal dilation of the aorta with an aortic diameter of 30 mm or greater is defined as an aortic aneurysm [1], [2]. Aortic aneurysms located under the diaphragm are classified as abdominal aortic aneurysms (AAA), occur more frequently than thoracic aortic aneurysms, and are responsible for approximately 15,000 deaths yearly [3], [4]. AAAs are often asymptomatic, and the size of the aneurysm has been shown to correlate with the risk of rupture [5], [6], which has a mortality rate of 85–95% [7]. Deaths from ruptured aneurysms are most effectively minimized by timely detection, followed by surveillance and open or endovascular repair of the aneurysm [8]. Although prophylactic, open surgical repair (OSR) of AAAs using a prosthetic graft was shown to reduce the mortality to 2–6% [6], [7], data from meta-analyses studies showed that open repair for ruptured AAAs (rAAAs) had a mortality rate as high as 48.5% [9], [10]. Efforts to reduce surgical insult, mortality and morbidity associated with open repair led to the development of minimally invasive endovascular aneurysm repair techniques (EVAR) [11]–[13]. A number of studies have reported that EVAR was associated with improved post-operative mortality rates compared to OSR [14]–[18]. Interestingly, although recent large randomized trials showed that EVAR was associated with a significantly lower operative mortality compared to OSR, it was also associated with higher rates of graft-related complications, and reintervention [5], [15], [16], [19]. Retrospective data from the United States showed that the use of endovascular repair for rAAAs has significantly increased over the past 10 years and the use of OSR has decreased [20].

Although ethical considerations limit the randomization of patients for emergency procedures, there has been a recent focus on designing randomized trials to evaluate EVAR as an alternative to OSR in the treatment of rAAA patients. However, the benefits of using EVAR in these patients remain controversial. A meta-analysis of 23 studies which compared outcomes of OSR and EVAR for rAAAs concluded that EVAR was associated with a significant reduction in 30-day mortality and a reduction in the mean hospital stay [21]. However, the heterogeneity and associated bias in this study make these data difficult to interpret. EVAR was also shown to be feasible in patients with rAAAs who were unsuitable for OSR due to hemodynamic instability or morphologic criteria [22]. Other studies suggested that EVAR was not significantly superior to OSR for rAAAs [23]–[25]. Interestingly, clinical outcomes after EVAR were shown to be associated with gender, and women with rAAAs had a significantly lower survival after emergent EVAR compared to men [26]. Outcome was also associated with age except for patients who received elective EVAR [27].

Despite the volume of data comparing EVAR to open repair for elective as well as emergent procedures, there are wide variations in trial design between studies and the paucity of studies evaluating EVAR for emergent procedures could be due to practical and ethical considerations. In this meta-analysis, we reviewed studies comparing the clinical outcomes in patients who underwent emergent open surgical repair and those who underwent emergent endovascular repair for abdominal aortic aneurysms. We analyzed data from 18 studies which compared the peri-operative mortality and length of hospital stay between these two patient populations.

## Methods

### Study Selection and Search Strategy

In this meta-analysis, we performed a systematic analysis of randomized controlled trials, prospective studies (either single-center or multi-center) or retrospective multi-center studies (including analysis of a national registry or database, eg. Medicare), which compared outcomes between patients who received emergent EVAR with those who received emergent OSR. Inclusion criteria were 1) presence of ruptured or leaking AAA, 2) patients had interventions of OSR or EVAR, 3) The timing of intervention was at the time of the emergency, 4) Included studies had to compare outcomes between emergent open and endovascular approaches to ruptured AAA repair and 5) only English language publications were analyzed.

The exclusion criteria were 1) single-arm studies, 2) when the surgery was elective and not emergent, 3) publications which were Letters, Comments, Editorials or Case Reports, 4) retrospective, single-center studies, and 5) when the study did not provide numerical information for the targeted primary and secondary outcomes.

The Medline, Cochrane, EMBASE,and Google Scholar databases were searched (until Apr 30, 2013). Reference lists of relevant studies were manually searched with keywords including abdominal aortic aneurysm, emergent, emergency, rupture, ruptured, leaking, leak, acute, endovascular, stent, graft, and endoscopic. Studies were identified by two independent reviewers based on the above criteria. Where there was uncertainty regarding eligibility, a third reviewer was consulted.

### Data Extraction

The following information/data were extracted from studies that met the inclusion criteria: the name of the first author, year of publication, study design, number of participants in each treatment group, participants’ age and gender, mortality, and length of hospital stay.

**Figure 1:**
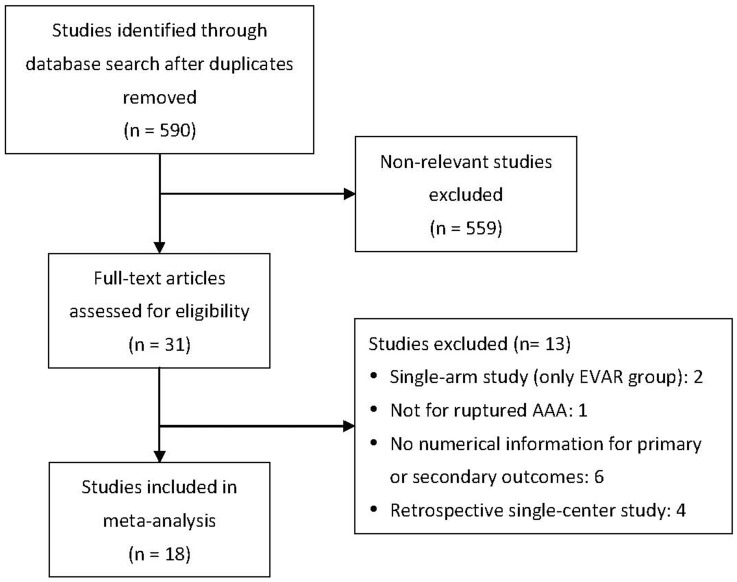
Flow chart for study selection. Abbreviation: EVAR, endovascular aneurysm repair; AAA, abdominal aortic aneurysm.

After reviewing full text articles, we excluded 13 studies based on exclusion criteria listed in Figure 1.

### Quality Assessment

The quality of primary studies was evaluated using the Newcastle-Ottawa Scale, which is a validated technique to assess the quality of nonrandomized studies [28]. The assessed outcomes for the 18 studies included in this meta-analysis are listed in Table 1.

**Table 1:** Characteristics of studies included in the meta-analysis.

Author (Year)	Study type	Institute or database	Number of cases	Age (year), mean	Gender, male (%)	Hospital stay (days), mean	Peri-operative mortality (%)	Conversion to open surgery (%)	Study quality (maximum 9)‡
Alsac JM (2005) [22]	prospective	Henri-Mondor University Hospital, Créteil (France)	17 vs. 20	72.9 vs. 72.8	94 vs. 100	Median: 11.5 vs. 20	23.5 vs. 50.0	17.6	8
Davenport DL (2010) [30]	retrospective	American College of Surgeons National Surgical Quality Improvement Program database (USA)	99 vs. 328	72.1 vs. 73.6	80 vs. 77	NA	22.2 vs. 37.2	NA	8
Egorova NN (2011) [26]	retrospective	Medicare Inpatient Standard Analytical and Denominator files (USA)	2007 vs. 46858	Male: 77.5 vs. 75.9	75 vs. 77	NA	41.1 vs. 52.7	7	7
				Female: 78.8 vs.:78.2					
Giles KA (2009) [31]	retrospective	Healthcare Cost and Utilization Project Nationwide Inpatient Sample database (USA)	2323 vs. 26106	Median: 75 vs. 73	NA	Median: 7 vs. 9	32.6 vs. 41.5	NA	8
Giles KA (2009) [32]	retrospective	American College of Surgeons National Surgical Quality Improvement Program database (USA)	121 vs. 446	72.6 vs. 73.7	80 vs. 76	Median: 7 vs. 10	24.0 vs. 36.0	4.1	8
Greco G (2006) [20]	retrospective	The hospital discharge databases for the four states of California, Florida, New Jersey and New York (USA)	NA	NA	NA	13.4 vs. 19	39.3 vs. 47.7	N = 20	8
Hinchliffe RJ (2006) [23]	Prospective, randomized controlled	University Hospital, Nottingham (UK)	15 vs. 17	Median: 74 vs. 80	73 vs. 76	Median: 10 vs. 12	53.3 vs. 52.9†	13.3	N/A
Holt PJ (2010) [33]	retrospective	National Health Service Hospital Episode Statistics (UK)	335 vs. 4079	NA	NA	NA	32.2 vs. 47.4	NA	7
Vogel TR (2009) [34]	retrospective	Healthcare Cost and Utilization Project New Jersey State Inpatient Database (USA)	82 vs. 618	NA	NA	14.08 vs. 13.42	45.1 vs. 52.4	NA	8
Leon LR Jr (2005) [27]	retrospective	Illinois Hospital Association COMPdata database (USA)	55 vs. 2063	NA	75 vs. 77	11.7 vs. 13.1	36.4 vs. 42.4	NA	8
Lesperance K (2008) [35]	retrospective	Healthcare Cost and Utilization Project Nationwide Inpatient Sample database (USA)	949 vs. 8982	73.9 vs. 73.1	78 vs. 76	Median: 6 vs. 9	31.0 vs. 42.0	NA	8
Mandawat A (2012) [36]	retrospective	Healthcare Cost and Utilization Project Nationwide Inpatient Sample database (USA)	64 vs. 207	70.5 vs. 72	73 vs. 76	NA	<18.0 vs. 35.7	NA	8
McPhee J (2009) [37]	retrospective	Healthcare Cost and Utilization Project Nationwide Inpatient Sample database (USA)	3179 vs. 24571	74.3 vs. 73	77 vs. 77	NA	31.7 vs. 40.7	NA	7
Park BD (2013) [38]	retrospective	Healthcare Cost and Utilization Project Nationwide Inpatient *Sample database (USA)	3796 vs. 12761	NA	75 vs. 75	9.91 vs. 13	27.4 vs. 41.0	0.64	8
Peppelenbosch N (2006) [24]	prospective	The New ERA trial; ten hospitals in Europe and Canada	49 vs. 51	75.1 vs. 73.8	86 vs. 80	14.9 vs. 22.2	35.0 vs. 39.0	6.1	8
Reimerink JJ (2013) [39]	Prospective, randomized controlled	Academic Medical Center, Amsterdam (The Netherlands)	57 vs. 59	74.9 vs. 74.5	86 vs. 85	Median: 9 vs. 13	21.0 vs. 25.0†	8.8	N/A
		VU University Medical Center, Amsterdam(The Netherlands)							
		OnzeLieveVrouweGastnhuis, Amsterdam (The Netherlands)							
Ten Bosch JA (2012) [18]	prospective	Catharina Hospital, Eindhoven (The Netherlands)	25 vs. 104	72.2 vs. 73.7	88 vs. 89	Median: 9.5 vs. 16	20.0 vs. 45.2	NA	7
Visser JJ (2009) [45]	prospective	Atrium Medical Center, Heerlen (The Netherlands)	58 vs. 143	73.2 vs. 73.5	93 vs. 83	NA	26.0 vs. 40.0	15.5	8
		Catharina Hospital, Eindhoven (The Netherlands)							
		Erasmus Medical Center, Rotterdam (The Netherlands)							
		Medical Spectrum Twente, Enschede (The Netherlands)							
		Medical Center Rotterdam Zuid, Rotterdam (The Netherlands)							
		University Medical Center, Groningen (The Netherlands)							
		University Medical Center, Nijmegen (The Netherlands)							

† The peri-operative mortality for these studies was calculated on an intention to treat basis.

‡ Study quality assessment performed using the Newcastle-Ottawa Scale.

Abbreviation: EVAR, endovascular repair; OSR, open surgical repair; AAA, abdominal aortic aneurysm.

### Outcome Measures

The primary outcome in this study was peri-operative mortality, which included intra-operative mortality, in-hospital mortality, and 30-day mortality. The secondary outcome was length of hospital stay.

### Statistical Analysis

The two outcomes used to compare the efficacy of the two surgical approaches in this meta-analysis were 1) peri-operative mortality and 2) length of hospital stay. Odds ratios (OR) with 95% confidence intervals (CI) were calculated for comparisons of peri-operative mortality rates between patients with rAAAs who underwent EVAR and those who underwent OSR; OR <1 indicates that the EVAR approach is favored. Difference in mean length of hospital stay with 95% CI was calculated for patients who underwent EVAR compared to those who underwent OSR. To analyze studies for which calculation of mean ± standard deviations (SD) was not possible, the mean and variance were estimated from the median, range, and sample size if these data were available [29]. Heterogeneity among the studies was assessed by calculating Cochran Q and the I^2^ statistic. For the Q statistic, P<0.10 indicated statistically significant heterogeneity. The I^2^ statistic indicates the percentage of the observed between-study variability caused by heterogeneity. Heterogeneity determined using the I^2^ statistic was defined as follows: 0 to 24% = no heterogeneity; 25 to 49% = moderate heterogeneity; 50 to 74% = large heterogeneity; and 75 to 100% = extreme heterogeneity. If either the Q statistic (P<0.1) or I^2^ statistic (>50%) indicated that heterogeneity existed between studies, the random-effects model (DerSimonian-Laird method) was used. Otherwise, the fixed-effects model was used (Mantel-Haenszel method). Pooled OR or difference in means was calculated and a 2-sided P value <0.05 was considered statistically significant. Sensitivity analysis was performed for primary outcome based on the leave-one-out approach. Publication bias was assessed by constructing funnel plots for primary outcome and quantitatively detected by fail-safe N (NFS) and Egger’s test. The absence of publication bias is indicated by the data points forming a symmetric funnel-shaped distribution, large NFS and P>0.10 in Egger’s test. All statistical analyses were performed using the statistical software Comprehensive Meta-Analysis, version 2.0 (Biostat, Englewood, NJ, USA).

## Results

### Study Characteristics

The process used for selection of studies is depicted in Figure 1. This meta-analysis included a total of 18 studies and the basic characteristics of the studies are summarized in Table 1 [18], [20], [22]–[24], [26], [27], [30]–[40]. Of the 18 studies, there were 2 randomized controlled trials, 4 prospective studies, and 12 retrospective studies. A total of 140,707 patients with rAAA were enrolled, of which 13,231 patients underwent EVAR and 127,476 underwent OSR. The total number of patients in each of studies ranged from 32 to 48,865. In the group that underwent EVAR, peri-operative mortality ranged from 20.0% to 53.3% in the group that underwent EVAR and from 25.0% to 52.9% in the group that underwent OSR. In general, patients who underwent EVAR had a shorter mean or median length of hospital stay compared to those who underwent OSR.

### Primary Outcome: Peri-operative Mortality

**Figure 2:**
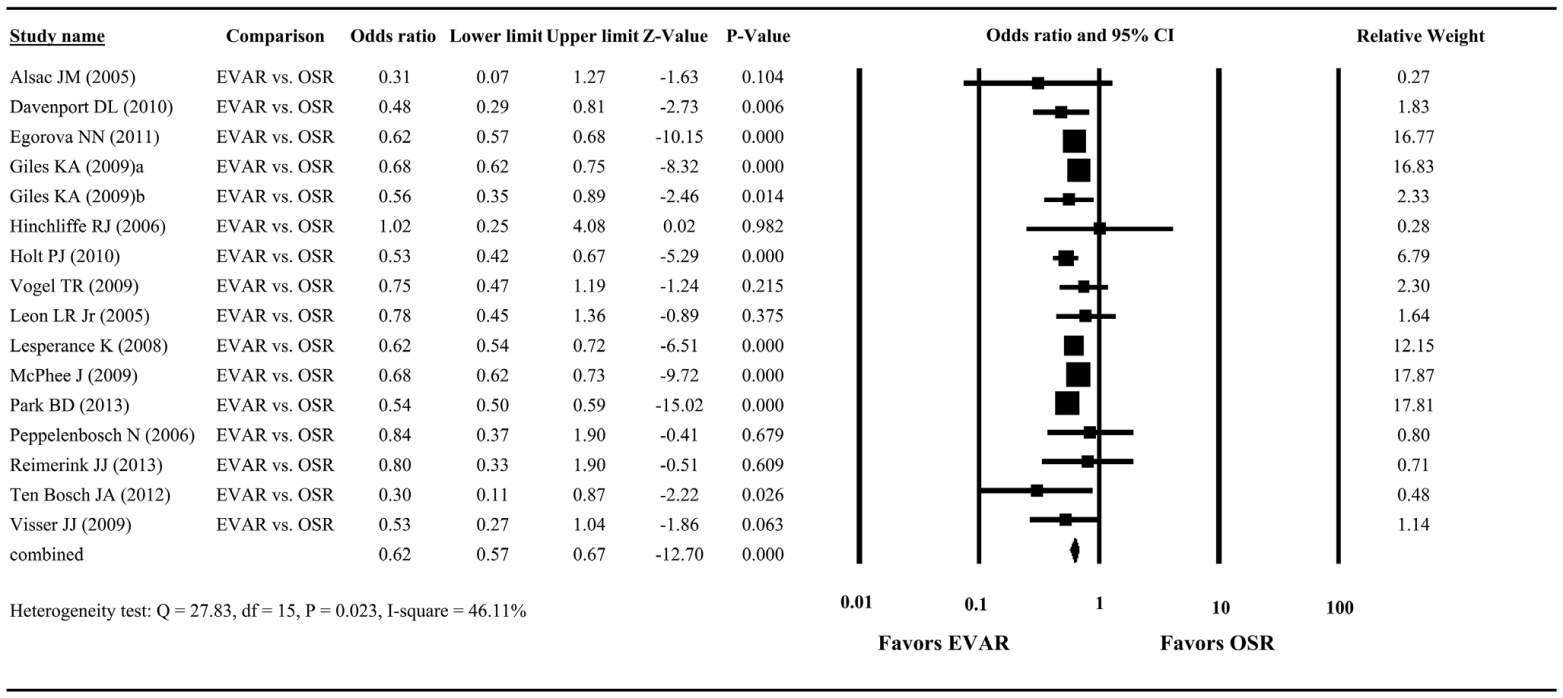
Forest plot showing results for the meta-analysis of peri-operative mortality: rAAA patients with EVAR vs. rAAA patients with OSR. Abbreviation: EVAR, endovascular aneurysm repair; OSR, open surgical repair; CI, confidence interval.

Since two of the selected studies did not have clear information regarding sample size [20] and peri-operative mortality [36], we only included a total of 16 studies in the meta-analysis. After pooling of data, we found significant heterogeneity among the studies (Q = 27.83, df = 15, P = 0.023; I^2^ = 46.11%), making it necessary to use a random-effects model for the meta-analysis of peri-operative mortality. The combined OR showed significantly lower peri-operative mortality in patients with EVAR compared to those with OSR. Among the 16 studies, ORs ranged from 0.30 to 1.02, with the overall OR being 0.62 (95% CI = 0.57 to 0.67, P<0.001, Figure 2).

### Secondary Outcome: Length of Hospital Stay

**Figure 3:**
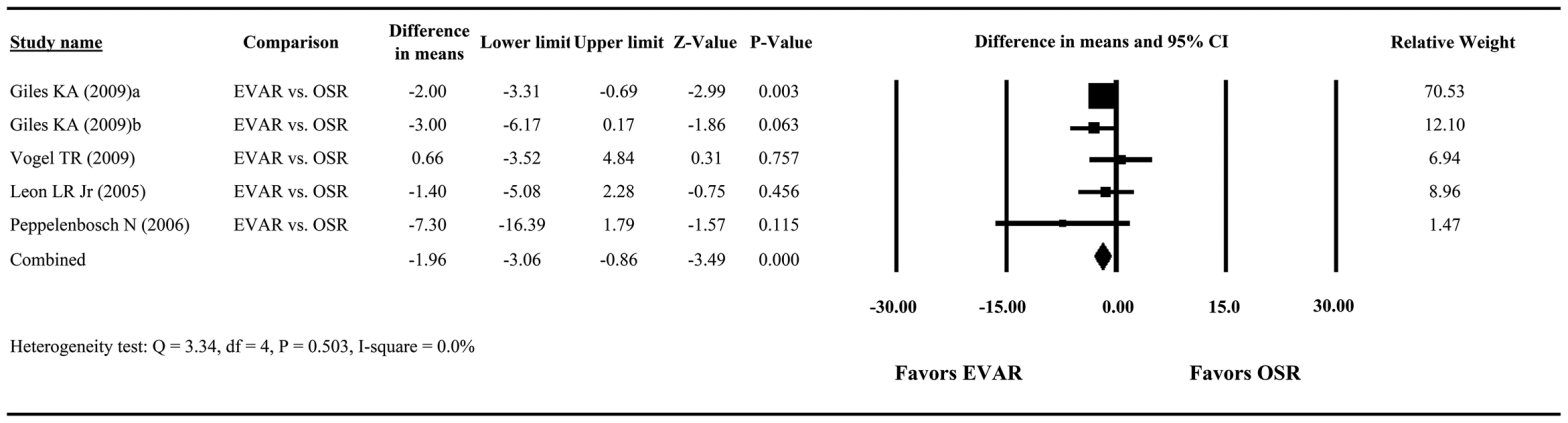
Forest plot showing results for the meta-analysis of length of hospital stay in rAAA patients with EVAR vs. rAAA patients with OSR. Abbreviation: EVAR, endovascular aneurysm repair; OSR, open surgical repair; CI, confidence interval.

Only 5 studies provided sufficient information to calculate the difference in mean length of hospital stay between the EVAR and OSR groups: two studies had mean ± SD for each group [[24], [27]], while three studies estimated the mean and variance using the median, range, and sample size [[31], [32], [41]]. After pooling of data, we did not find a significant heterogeneity among the studies (Q = 3.34, df = 4, P = 0.503; I^2^ = 0.0%) and therefore used a fixed-effects model for the meta-analysis of the length of hospital stay. The combined difference in means showed a significantly shorter mean length of hospital stay in patients with EVAR compared to those with OSR. Among the 5 studies, the difference in mean length of stay ranged from −7.30 to 0.66 days, with the overall estimate being −1.96 days (95% CI = −3.06 to −0.86, P<0.001, Figure 3).

### Sensitivity Analysis

**Figure 4:**
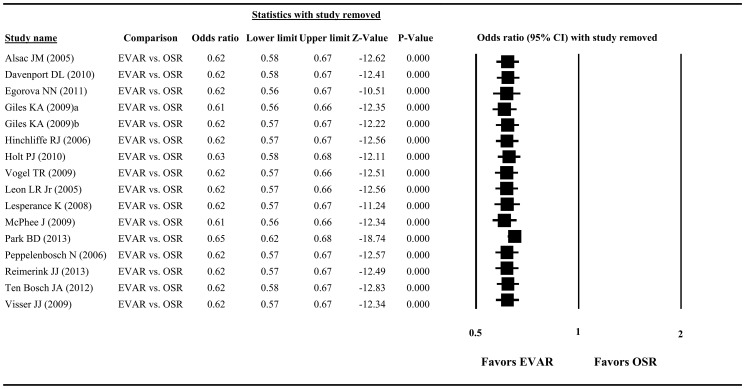
Results of sensitivity analysis to examine the influence of individual studies on pooled estimates of peri-operative mortality as determined by the “leave-one-out” approach. Abbreviation: EVAR, endovascular aneurysm repair; OSR, open surgical repair; CI, confidence interval.

The results of sensitivity analysis, in which studies were omitted one at a time, are summarized in Figure 4. For peri-operative mortality, the direction and magnitude of the pooled estimate did not vary markedly with the removal of any study (Figure 4), indicating good reliability in this meta-analysis.

### Publication Bias

**Figure 5:**
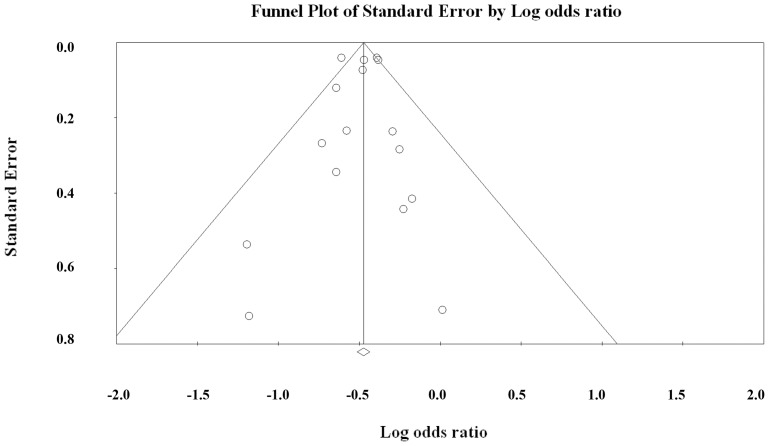
Funnel plot for evaluating publication bias while reporting peri-operative mortality. White circles represent published article and white rhombuses represent the actual combined effect sizes, respectively.

The funnel plot for publication bias (standard error by log odds ratio of peri-operative mortality) demonstrated evidence of symmetry (Figure 5), indicating no evidence of publication bias. Additionally, the combined effect size for the 16 studies yielded a Z value of −17.23 and corresponding 2-tailed p-value of <0.0001. The NFS was 1221, indicating that we would need to locate and include 1221 ‘null’ studies in order for the combined 2-tailed p-value to exceed 0.050. We also used the Egger’s test to show that there was no publication bias for peri-operative mortality (Figure 5, t = 0.345, P = 0.368).

## Discussion

In this meta-analysis of 18 studies, we compared peri-operative mortality and length of hospital stay in patients with rAAAs who underwent emergent EVAR to those who underwent emergent OSR. Our analysis showed that patients who underwent emergent EVAR had significantly lower peri-operative mortality rates and a significantly shorter hospital stay compared to those who underwent emergent OSR.

The use of EVAR for rAAAs has increased in the United States, especially in urban areas, likely because EVAR is associated with reduced mortality and a reduced complication rate compared to OSR [38], [42]. However, there are conflicting data on the efficacy and outcomes of EVAR in patients with rAAAs due to wide variations in trial design. Data from some of the retrospective studies included in our analysis showed that 1) mortality rates have decreased in patients receiving emergent EVAR for rAAAs, while the rates have remained stable in patients who received emergent OSR, 2) high-volume institutions have lower mortality rates compared to low-volume institutions [31], [35], [37] and 3) in-hospital mortality for emergent AAA repairs is lower in hospitals with a greater number of elective AAA repairs, possibly due to the higher numbers of specialist surgeons and vascular critical care facilities [33].

The ACS National Surgical Quality Improvement Program (ACS NSQIP) is the first nationally validated, risk-adjusted, outcomes-based program to measure and improve the quality of surgical care (http://www.facs.org/cqi/outcomes.html). Some retrospective studies analyzing the NSQIP database showed a significantly lower composite 30-day morbidity risk and a lower intraoperative transfusion requirement in patients with rAAAs after EVAR compared to open repair [30], [32]. Data from two retrospective studies included in our analysis showed that 1) rAAA patients who received EVAR had a significant survival benefit compared to patients who received OSR [33] and 2) EVAR resulted in superior short-term outcomes compared to open repair in rAAA patients who are transferred to institutions with EVAR facilities [36]. Peppelenbosch et al. showed lower first-month mortality and lower blood loss in a prospectively enrolled group of patients with rAAAs when compared to a retrospective control group [41]. These studies were consistent with results from a recent meta-analysis of randomized and risk-adjusted observational studies comparing EVAR vs. open repair for rAAA, which showed that EVAR was associated with a significantly lower mortality rate compared to open repair [43]. Another recent meta-analysis of 41 studies showed that emergent EVAR was associated with a significantly lower risk of in-hospital mortality and respiratory, renal and cardiac complications compared to OSR [44].

However, these data contrast with studies showing that although patients who received emergent EVAR had better 30-day mortality rates compared to patients who received OSR, this was not statistically significant [39], [40]. Data from a randomized controlled trial included in our analysis also did not show a significant benefit conferred by EVAR, which could be due to the small sample size of this study [23]. Additionally, a retrospective cohort study included in our analysis showed no survival benefit in patients who received emergent EVAR compared to those who received OSR. This was suggested to be due to a number of factors including the lack of well-established EVAR facilities and patient insurance constraints [34]. Our meta-analysis included data from a randomized trial in Amsterdam which showed no significant difference in combined death or severe complications between patients who received emergent EVAR and those who received OSR. This was attributed to optimization of logistics and protocols, resulting in comparable outcomes with both procedures [39]. Although our data showed an overall benefit in terms of peri-operative mortality and length of hospital stay in rAAA patients compared to OSR, there is an urgent need to resolve such discrepancies with prospective randomized controlled trials performed on larger sample sizes.

It is important to note that in our present meta-analysis of 18 studies, only 7 were prospective studies and only 2 were randomized, controlled studies. Although we included multi-center, retrospective studies, we excluded single-center, retrospective studies in order to prevent selection bias. We are also aware that inclusion of two large population-based studies in this meta-analysis [26], [37] is a limitation of this study, since they may incorporate duplications. The small number of qualified studies could reflect the practical and ethical considerations which limit the design of randomized, controlled trials to compare EVAR and OSR for ruptured AAAs. These considerations include the endovascular expertise of the surgeons, the time required for a CT scan prior to EVAR and the time required for randomization [23], [39]. However, since it was shown that CT scanning did not delay treatment, it is possible to recruit patients with ruptured AAAs to randomized trials to compare outcomes of EVAR and OSR [23]. After pooling data from 16 studies, we showed significantly lower peri-operative mortality rates in rAAA patients who received emergent EVAR compared to those who received OSR. We also used a random-effects model to analyze data from 5 studies and showed that rAAA patients who received EVAR had significantly shorter hospital stays compared to those who received OSR. It is possible that our data could be a reflection of the fact that emergent EVAR attenuates the inflammatory response, which could be advantageous in rAAA patients [21].

Dropping one study at a time from the meta-analysis did not significantly change the direction or magnitude of the pooled data, indicating the reliability of this analysis. We also used a funnel plot and the Egger’s test to show that there was no publication bias in our meta-analysis.

In summary, we showed that EVAR conferred significant benefits in terms of peri-operative mortality and length of hospital stay in rAAA patients compared to OSR. A number of factors such as logistics and preoperative CT scanning could explain the dearth of prospective randomized controlled trials to compare EVAR and OSR in rAAA patients.

## Supporting Information

Checklist S1 (DOC)
